# Catheter balloon mimicking incarcerated femoral hernia and co-existing small bowel diverticular perforation: a case report

**DOI:** 10.4076/1757-1626-2-8755

**Published:** 2009-09-15

**Authors:** Katherine Reeve, Alex Hotouras, Muralidharan Manghat, Suresh Pillai

**Affiliations:** 1 Department of General Surgery and Colorectal Surgery, Lincoln County Hospital, Lincoln, UK

## Abstract

The majority of patients with small bowel diverticula are asymptomatic, however, associated complications include inflammation, intestinal obstruction, perforation and gastrointestinal haemorrhage. Bladder divertulae are uncommon and can herniate into the femoral or inguinal canal as well as the scrotum. We report the case of an elderly lady who underwent laparotomy for an incarcerated femoral hernia and was found to have the catheter balloon stuck into a bladder diverticulum in the femoral canal and coexisting small bowel diverticular perforation.

## Case presentation

**Figure 1:**
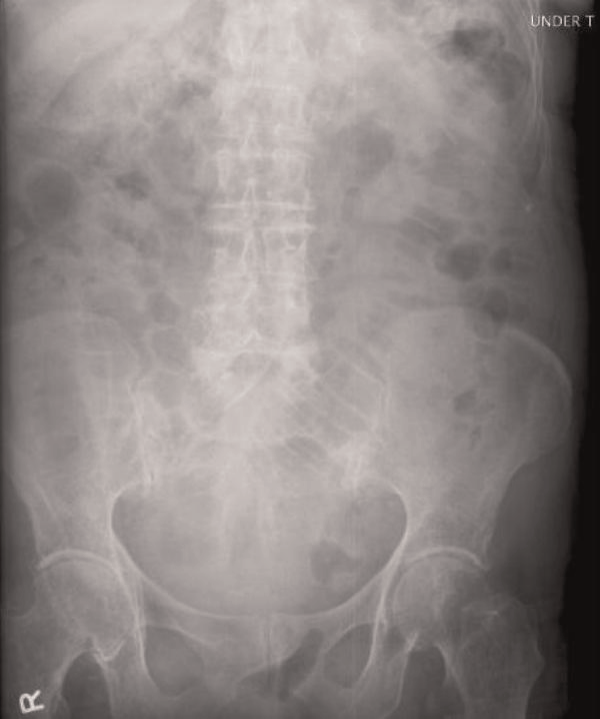
**Plain radiograph of the abdomen**.

**Figure 2:**
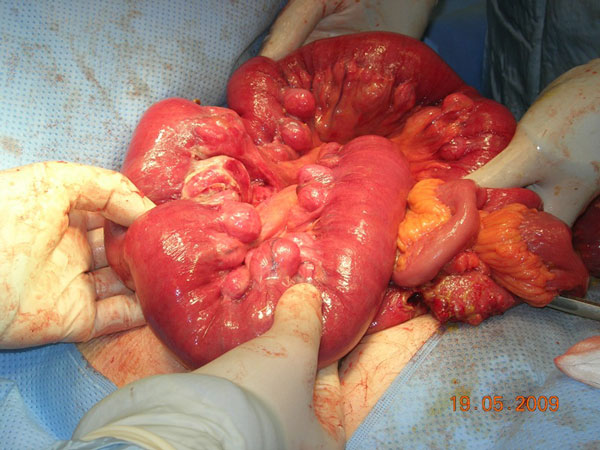
**Photograph displaying jejunal diverticular disease at operation**.

A slender 87-year-old Caucasian lady presented with a five day history of colicky generalised abdominal pain and not opening her bowels. She was nauseated and vomited on a few occasions. She had no history of bleeding per rectum or change in bowel habit but she reported a one and a half stone weight loss over twelve months. She also described symptoms of urinary frequency. Her past medical history consisted of angina, valvular disease and an abdominal hysterectomy and salpingo-oophorectomy 20 years ago for post-menopausal bleeding with no evidence of malignancy. She was a non-smoker and consumed approximately seven units of alcohol per week. On clinical examination, there was tenderness in the right iliac fossa with generalised abdominal distension, bowel sounds were present and there was no guarding or rebound tenderness. The white cell count was normal at 7.9 × 109/L and the C-reactive protein titre was 37 mg/L. A plain abdominal radiograph displayed proximal small bowel loops and air in the large bowel (Figure 1). A provisional diagnosis of small bowel obstruction secondary to adhesions or malignancy was made. She was treated conservatively with a nasogastric tube, catheterisation, intravenous fluids and kept nil by mouth. On review the following morning, the patient was complaining of a tender swelling in the right groin and increasing abdominal pain. A diagnosis of an incarcerated femoral hernia was made and the patient was taken to theatre for repair of her femoral hernia. Examination of the abdomen while the patient was on the operating table showed that the swelling had disappeared. A decision for a lower midline laparotomy was taken. At laparotomy, extensive diverticulosis of the jejunum was present with a diverticular stricture being the cause of small bowel obstruction and subsequent perforation (Figure 2). The diseased segment of small bowel was resected with an end-to-end anastomosis. In addition, a small cystic swelling was found near the femoral canal. Careful inspection confirmed this to be the balloon of the Foley catheter extending from a bladder diverticulum. The patient made a good recovery post-operatively and was discharged home 3 weeks later.

## Discussion

Small bowel obstruction has many causes including adhesions, hernias, malignancy and inflammatory bowel disease. A common cause of small bowel obstruction and bowel strangulation is femoral herniation of the small bowel [1]. Hernias have been found to be the most common cause of bowel strangulation [2]. Extremely rarely diverticular disease of the small bowel may be complicated by small bowel obstruction.

Due to the rarity of small bowel diverticular complications, this is not the first diagnosis entertained for a patient presenting with obstruction, bleeding or an acute abdomen. This can lead to some delay in diagnosis and definitive treatment. The most useful imaging technique for diagnosing small bowel diverticulosis is CT scanning, however the disease often only presents at laparotomy [3]. Small bowel diverticula commonly occur in the duodenum, however only around 2% of the population have jejunal diverticula [4,5]. Jejunal diverticula have a higher rate of complication than duodenal diverticula, suggesting that pre-emptive surgical removal on discovery may be preferable in comparison to conservative management [5]. The formation of strictures secondary to jejunal diverticular disease has previously been reported to be complicated by small bowel obstruction [6]. One of the many complications of small bowel diverticula, along with obstruction, includes perforation. This is generally treated surgically with laparotomy and bowel resection. However, non-surgical management is a further option with either sole antibiotic treatment or diverticular-associated abscess drainage [7].

Herniation of the urinary bladder wall is uncommon. The incidence of groin hernia containing the urinary bladder has been shown to be around 0.36%, although higher incidences have been reported [8]. Often such hernias can be associated with iatrogenic bladder injury at the time of surgery. Bladder herniation most commonly occurs through the inguinal and femoral canals. Causal factors of bladder herniation include pelvic space-occupying lesions, urinary outlet obstruction and obesity leading to increased intra-abdominal pressures.

A high index of suspicion of the content of groin hernia is required to avoid iatrogenic injury to abdominal organs. Although unusual contents of groin hernias, such as the bladder, can be picked up by imaging, such as excretory urography, the likelihood is that the contents are only delineated at surgery [9]. From review of the literature, no examples of urinary catheters housed in bladder diverticulae have been the cause of supposed incarcerated femoral hernias prior to this case.

## Consent

Written informed consent was obtained from the patient for publication of this case report and accompanying images. A copy of consent is available for review by the Editor-in chief of this journal.

## Competing interests

The authors declare that they have no competing interests.

## Authors' contributions

AH, MM and SP all performed the initial clinical assessment of the patient and surgical procedure. KR collected patient data and researched the literature for discussion. KR and AH formulated a draft report. All authors contributed in the final report.
